# Ventromedial hypothalamic nucleus in regulation of stress-induced gastric mucosal injury in rats

**DOI:** 10.1038/s41598-018-28456-0

**Published:** 2018-07-05

**Authors:** Haiji Sun, Pan Zhao, Wenkai Liu, Lei Li, Hongbin Ai, Xiaoli Ma

**Affiliations:** 1 Key Laboratory of Animal Resistance Biology of Shandong Province, School of Life Science, Shandong Normal University, Jinan, 250014 China; 2 Central Laboratory, Jinan Central Hospital Affiliated to Shandong University, Jinan, 250013 China

## Abstract

Previous studies showed that restraint water-immersion stress (RWIS) increases the expression of Fos protein in the ventromedial hypothalamic nucleus (VMH), indicating the VMH involving in the stress-induced gastric mucosal injury (SGMI). The present study was designed to investigate its possible neuro-regulatory mechanisms in rats receiving either VMH lesions or sham surgery. The model for SGMI was developed by restraint and water (21 ± 1 °C) immersion for 2 h. Gastric mucosal injury index, gastric motility, gastric acid secretion and Fos expression in the hypothalamus and brainstem were examined on the 15th postoperative day in RWIS rats. Gastric mucosal injury in VMH-lesioned rats was obviously aggravated compared to the control. Gastric acidity under RWIS was obviously higher in VMH-lesioned rats than that in sham rats. Meantime, the VMH-lesioned rats exhibited marked increases in the amplitude of gastric motility in the VMH lesions group after RWIS. In VMH-lesioned rats, Fos expression significantly increased in the dorsal motor nucleus of the vagus (DMV), the nucleus of the solitary tract (NTS), the area postrema (AP), the paraventricular nucleus (PVN) and the supraoptic nucleus (SON) in response to RWIS. These results indicate that VMH lesions can aggravate the stress-induced gastric mucosal injury through the VMH-dorsal vagal complex (DVC)-vagal nerve pathway.

## Introduction

The stress-induced gastric mucosal injury(SGMI)occurs in a wide variety of stress situation, including severe traumatic injury, various operations and sepsis, etc^1^. Recently, studies have indicated that SGMI is closely related to gastric acid, pepsin secretion, gastric mucosal blood flow, prostaglandins, reactive oxygen species and gastric mucosal cell proliferation^2–4^. However, the role of the central nuclei in regulating SGMI is far from clear.

In the hypothalamus, the ventromedial hypothalamic nucleus (VMH) is an important complex nucleus that receives various inputs and directly projects to the brainstem and spinal cord^5,6^. It is known that the VMH is associated with the regulation of feeding behavior and satiety. Bilateral VMH lesions led to hyperphagia and obesity, while electrical stimulation of the neurons in the VMH cause the opposite effect^7,8^. Some studies have shown that the VMH is involved in response to stress, including using the activity–stress paradigm, resulting in excessive running and stomach ulceration^9^, enhancing a variety of nondefensive behaviors to both cat odor and foot shock after VMH neurotoxic lesion, and mediating immobilization (IMB) stress-induced hypocalcemia via its influence on the gastric vagus^10^.

The mutual influence of the gastrointestinal tract and central nervous system is called “gut-brain axis”, which plays a potential role in numerous physiological and pathological conditions^11^. It is known that stomach dysfunction could be a risk factor for behavioral disorders in rats. Moreover, psychological distress is associated with functional gastrointestinal disorders. Restraint water-immersion stress (RWIS) has been widely accepted for studying the mechanisms of stress-induced gastric mucosal injury^12^. In our previous study, the results demonstrated that RWIS induced remarkable Fos expression in the VMH, indicative of increased neuronal activity, but the role of VMH in the genesis of stress-induced gastric mucosal injury is unclear. The PVN is a neuronal nucleus in the hypothalamus and is activated by a variety of stressful changes, including RWIS^13,14^. The PVN and VMH are strongly connected with each other and involved in stress-related behaviors^15^. However, during RWIS, the relationship between the PVN and VMH is still unknown. In addition, our results have demonstrated that RWIS results in increased levels of Fos protein expression in the DMV, NA, NTS and AP^13^. Neuronal hyperactivity of the dorsal vagal complex (DVC), which is comprised of the DMV, NTS, and AP, caused gastric lesions^16,17^. Electrical and chemical stimulation of the DVC decreased gastric motility, and enhanced the volume of gastric juice^18^. Electrophysiological studies have demonstrated that cells in the VMH can modulate the activity of neurons in the DVC. So far, these results lead us to hypothesize that the excitability of VMH neurons may be involved in the stress-induced gastric mucosal injury through the DVC-vagal nerve pathway. In this study, we measured the gastric mucosal injury index, gastric motility, gastric acid secretion and Fos expression in the PVN, SON, DMV, NTS and AP after VMH lesions in RWIS rats.

## Material and Methods

### Animals

Male Wistar rats weighing 180–220 g were purchased from the Experimental Animal Center of Shandong University (Jinan, China)and housed two per cage in a room at temperature (22 ± 2 °C). They were kept under a normal day/night cycle for at least 7 days before the experiments. The care and handing of the animals were in according with the National Institutes of Health guidelines and approved by the Experimental Animal Ethics Committee of Shandong Normal University (Jinan, China).

### Experimental design

Three sets were prepared in both VMH-lesioned rats (lesion group) and sham VMH-lesioned rats (control group) (n = 7 per group; n = 42 total) to evaluate the role of the VMH nucleus in SGMI. Three sets were as follows: rats were used to record gastric motility in RWIS; rats were used to measure gastric acid secretion in RWIS; rats were used to measure gastric mucosal injury index and Fos expression in RWIS.

### VMH lesions

The rats were anesthetized with pentobarbital (70 mg/kg) and fixed in a stereotaxic apparatus (Stoelting 51600, USA) after an 18 h fast. An electrode (40–60 μm in diameter and 10–16 kΩ in resistance) was introduced with the following stereotaxic coordinates: 1.2 mm posterior to the bregma, 0.6 mm lateral to the midsagittal suture, and 9.9 mm below the surface of the skull. The electrolytic lesions were produced with an 1-mA current for 10 s in the both sides of VMH. The sham-lesioned rats were set up in an identical manner, but no current was passed through the needle. All operated animals were allowed to recover from surgery for 15 days before being used for studies. At the end of experiments, the brains of VMH-lesioned rats were removed, and neutral red staining was used to verify the success of the formation of VMH lesions. Body weights of the rats were measured at 0, 3, 6, 9, 12 and 15 days after VMH lesions.

### Water-restraint stress

The four limbs of rats were fixed on a wooden board with light ether anesthesia. After the rats were conscious, they were vertically immersed in water (21 ± 1 °C) to the level of the xiphoid for 2 h. Afterward, the rats were deeply anesthetized by overdose of pentobarbital sodium (100 mg/kg body weight).

### Gastric motility curves

A midline laparotomy was performed, and a latex balloon attached to a thin polyethylene tube was inserted into the pylorus through the forestomach and the polyethylene tube was connected to a pressure transducer. The stomach was inflated by introducing warm saline (0.5 ml to 1.0 ml) into the balloon to achieve a baseline pressure of 5 cm to 10 cm hydraulic pressure. Gastric motility curves were continuously recorded by a two-lead physiological recording instrument (LMS-2B, Chengdu Instrument Factory, China) for 2 h. To evaluate gastric motility, we considered two parameters: contraction frequency and amplitude of contraction under stress conditions. The MI was defined as the product of amplitude and duration of every contraction wave. Contraction frequency was defined as the mean of contractions per min and contraction amplitude was defined as the mean of contraction amplitude per min. To evaluate whether the effect of VMH lesion was time-dependent, gastric motility measurements at 3 intermittent ten min intervals (t1 = 20–30, t2 = 50–60 and t3 = 110–120 min) were chosen.

### Measurements of gastric acid secretion

The stomach was exposed by a midline laparotomy, and then the junction of the duodenum and stomach was also ligated. After RWIS, gastric cardia was also ligated. The stomach was removed, and gastric effluent was collected. Gastric juice output, titratable H+ quantum and H+ concentration were measured to study gastric secretion. Values for titratable H+ quantum were determined by titration of the flushed perfusate with 0.01 N of NaOH using an autotitrator (TTT80 Titrator, Radiometer, Copenhagen, Denmark).

### Assessment of gastric mucosal injury index

The stomachs were fixed with 10 ml of 1% Formalin after the rats were sacrificed. Each stomach was opened by cutting along the greater curvature, cleaned, and spread after 30 min. The stomach was examined with a binocular dissection microscope. Scores are counted as follows: 1 for a lesion ≤1 mm, 2 for a lesion >1 mm and ≤2 mm, and 3 for a lesion >2 mm and ≤3 mm. For lesions with a width >1 mm, the lesion score was doubled^17^.

### Immunohistochemistry

Frozen coronal sections were cut 30 mm thick. After being rinsed in 0.01 M of PBS, they were incubated with blocking buffer (5% goat serum and 0.3% Triton X-100 in PBS) for 30 min and then with rabbit anti-Fos antibody (sc-52, Santa Cruz, CA, USA) at a dilution of 1:2000 for 24 h at 44 °C. Subsequently, the sections were incubated with the biotinylated goat anti-rabbit IgG (Zymed Laboratories, San Francisco, CA, USA) for 1 h at room temperature. The specificities of the staining were tested on the sections in the control rats by omitting the primary antibodies.

### Statistical analysis

The data are expressed as the mean ± SEM. The statistical procedures were conducted using SPSS13.0 software (SPSS, Chicago, IL, USA). Comparisons between two groups were conducted using T-tests or a repeated measures one-way analysis of variance (ANOVA) with Dunnett’s post hoc test. A P-value < 0.05 was considered significant.

## Results

### Histological verification

**Figure 1:**
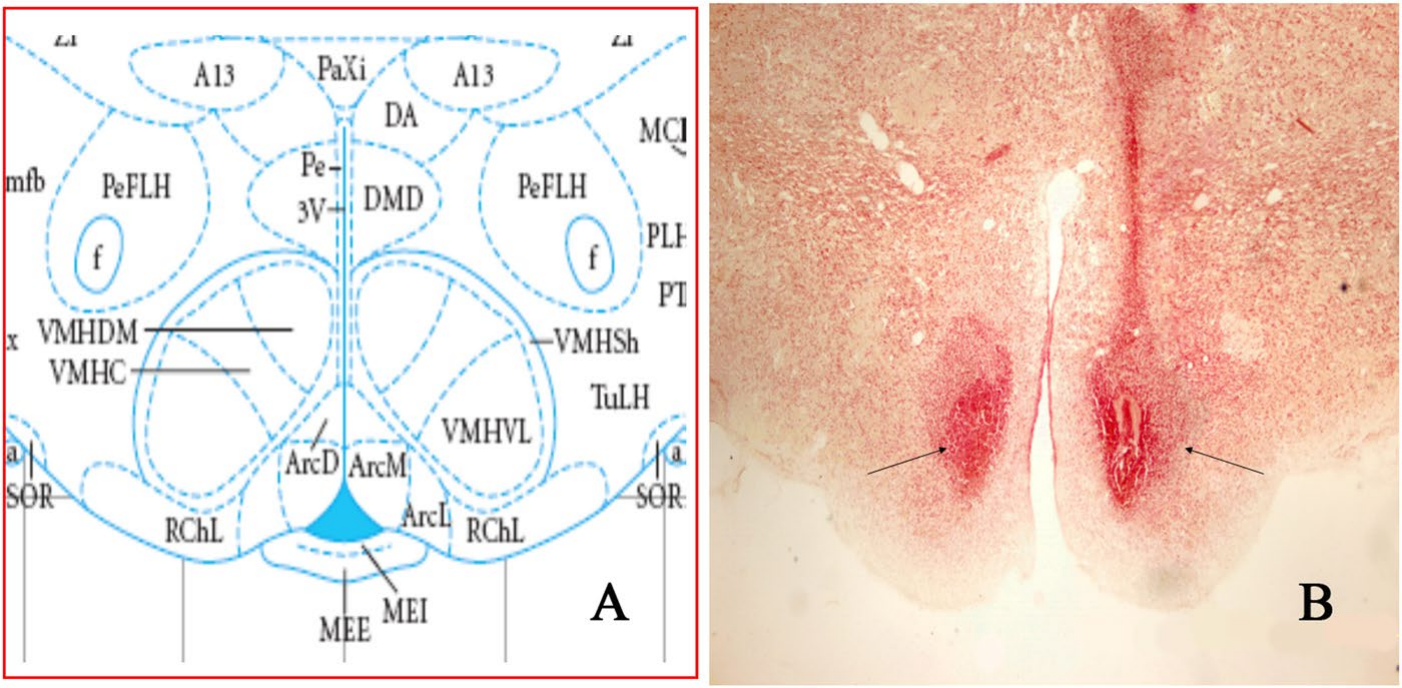
The target sites of the needle tip of electrolytic lesions in the VMH. (**A**) Standard atlas sections of the rat brain showing the distributions of electrolytic lesions sites for the experimental rats. (**B**) Photomicrographs of electrolytic lesions site in the rat brain.

The VMH, including its corresponding brain atlas, is presented in Fig. 1A. The electrolytic lesions were affirmed based on the Paxinos and Watson’s stereotaxic atlas (Paxinos and Watson, 1998) (Fig. 1A,B). Lesions were considered successful when more than 40% of the VMH was destroyed histologically. Such lesions were observed in 30.0% (21/70) of all lesioned rats.

### Body weight

The body weights of VMH-lesioned rats and sham rats were similar at 245.8 ± 14.7 and 258.1 ± 2.1, respectively, before VMH lesions (Table 1). During 12 days post surgery, the body weight increased daily in each of the two groups and was not significantly different between them. On the 15th day after VMH lesions, the average body weights were 330.6 ± 11.9 g and 289.2 ± 5.3 g, respectively, and VMH-lesioned rats were heavier than the sham rats (Table 1) (p < 0.05).

**Table 1:** Body weight levels in the sham and VMH-lesioned rats.

Group	Days after operation					
	0	3	6	9	12	15
Sham (n = 21)	258.1 ± 2.1	259.0 ± 5.3	268.5 ± 3.1	276.6 ± 2.2	283.6 ± 4.1	289.2 ± 5.3
VMH lesioned (n = 21)	245.8 ± 14.7	252.7 ± 15.7	263.2 ± 11.6	285.0 ± 13.5	304.3 ± 12.4	330.6 ± 11.9*

Mean ± SEM, *P < 0.05, Sham vs VMH-lesioned rats.

### Effects of VMH lesions on gastric mucosal injury induced by RWIS

**Figure 2:**
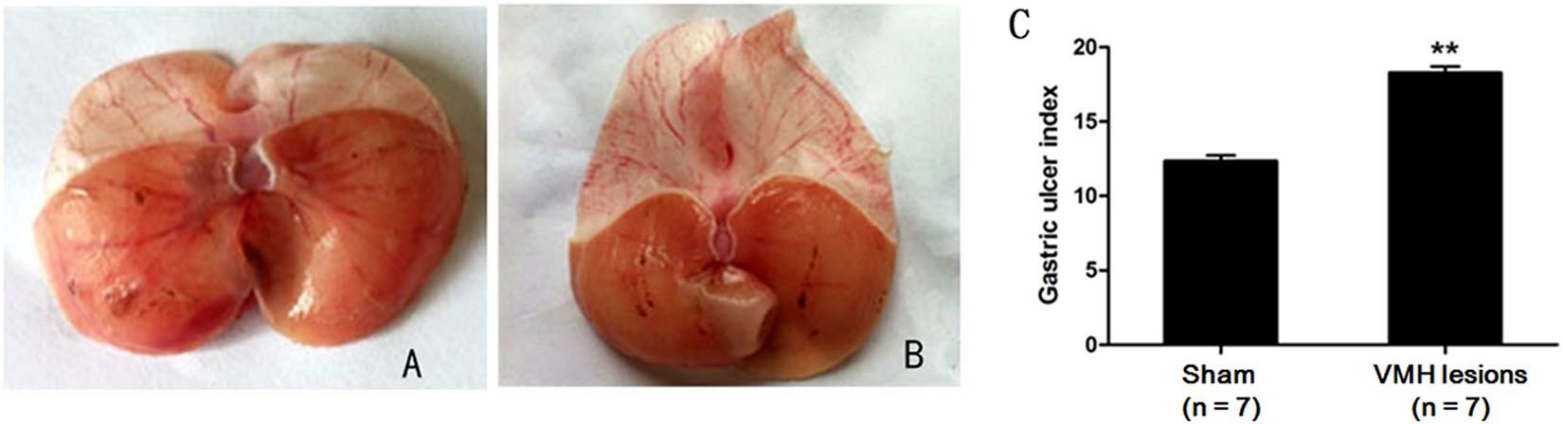
Effects of VMH lesions on gastric mucosal injury induced by RWIS. (**A**) Gastric mucosal injury in the sham group. (**B**) Gastric mucosal injury at VMH lesions. (**C**) The erosion index increased in VMH lesions. Data are presented as the means ± SEM (n = 7). **P < 0.01 vs sham group.

Water-immersion restraint stress induced gastric erosions both in the VMH-lesioned rats and in the sham VMH-lesioned rats. However, the extent of the damage differed (Table 2 and Fig. 2A,B). The VMH lesions potentiated stress-induced gastric erosions by 47.98%. The gastric mucosal damage index in VMH-lesioned rats (18.29 ± 1.11 was much higher than that in the sham-operated rats (12.36 ± 0.96), suggesting an aggravating effect of VMH lesions on stress-induced gastric mucosal injury.

**Table 2:** Effects of VMH lesions on gastric mucosal injury induced by RWIS.

	Gastric ulcer index	Change rate (%)	*P* value
Sham (n = 7)	12.36 ± 0.96		
VMH lesions (n=7)	18.29 ± 1.11^**^	47.98	0.002

Mean ± SEM, ^**^P < 0.01, Sham vs VMH-lesioned rats.

### Effects of VMH lesions on gastric acid secretion induced by RWIS

Table 3 shows the effects of VMH lesions on the volume of gastric juice, gastric acid output and gastric pH induced by RWIS. The volume of gastric juice and gastric acid output in VMH-lesioned rats was significantly higher than that in control rats, while the gastric pH in VMH-lesioned rats was lower compared with the control rats (Table 3).

**Table 3:** Effects of VMH lesions on gastric acid secretion induced by RWIS.

	Gastric juice (mL/2 h)	Acid output (μmol/2 h))	Gastric pH
Sham (n = 7)	0.57 ± 0.05	9.73 ± 1.25	1.97 ± 0.20
VMH lesioned (n = 7)	1.06 ± 0.14^*^	59.37 ± 5.12^*^	1.25 ± 0.07^**^
Change rate (%)	85.96	510.17	−36.55
*P* value	0.019	0.014	0.006

Mean ± SEM, ^*^P < 0.05,^**^P < 0.01, Sham vs VMH-lesioned rats.

### Effects of VMH lesions on gastric motility induced by RWIS

**Figure 3:**
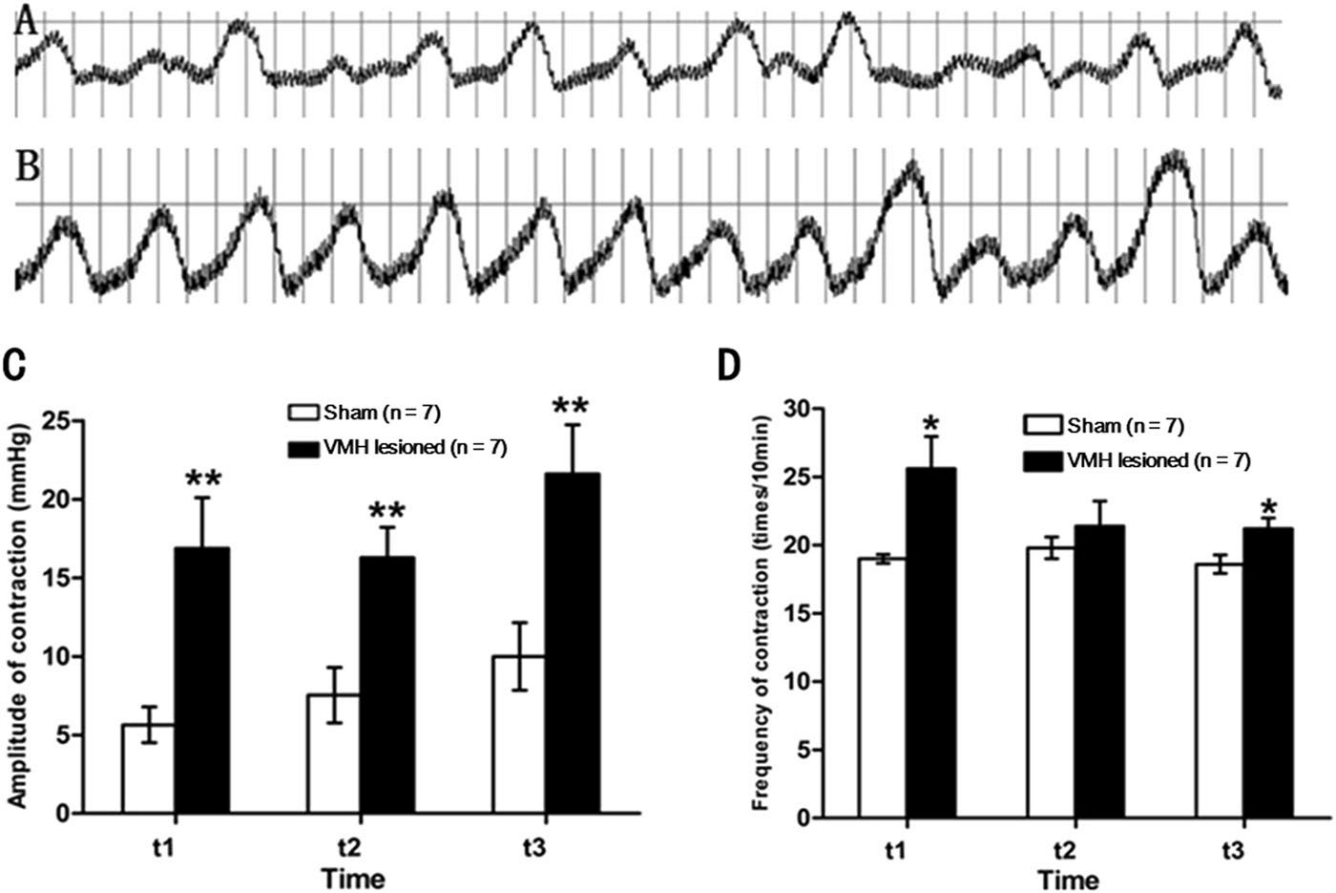
Effects of VMH lesions on gastric motility induced by RWIS. (**A**) Gastric induced motility by RWIS in sham rats. (**B**) Gastric motility induced by RWIS in VMH-lesioned rats. (**C**) Comparison of amplitude of gastric motility in sham VMH lesioned rats and in VMH-lesioned rats. (**D**) Comparison of frequency of gastric motility in sham VMH lesioned rats and in VMH-lesioned rats. Data are presented as the means ± SEM (n = 7). *P < 0.05, **P < 0.01 Sham vs VMH-lesioned rats.

Compared with the sham group, VMH lesions markedly increased the contraction amplitude of gastric motility induced by RWIS (16.3 ± 1.9 vs 7.5 ± 1.7, *P* = 0.01 in the first intermittent 10 min intervals; 21.6 ± 3.1 vs 10.0 ± 2.1, *P* = 0.01 in the second intermittent 10 min intervals; 31.4 ± 4.4 vs 13.8 ± 2.7, *P* = 0.01 in the third intermittent 10 min intervals (Fig. 3A–C). The contraction frequency was larger in VMH-lesioned rats compared with control rats: 21.20 ± 0.80 vs 118.60 ± 0.68, *P* = 0.04 in the first intermittent 10 min intervals, and 21.20 ± 0.80 vs 18.60 ± 0.68, P = 0.038 in the third intermittent 10 min intervals; however there was no difference between the VMH-lesioned group and the sham group in the second interval (Fig. 3A,B,D).

### Effects of VMH lesions on RWIS-induced neuronal activity in the PVN, SON, DMV, NTS and AP

**Figure 4:**
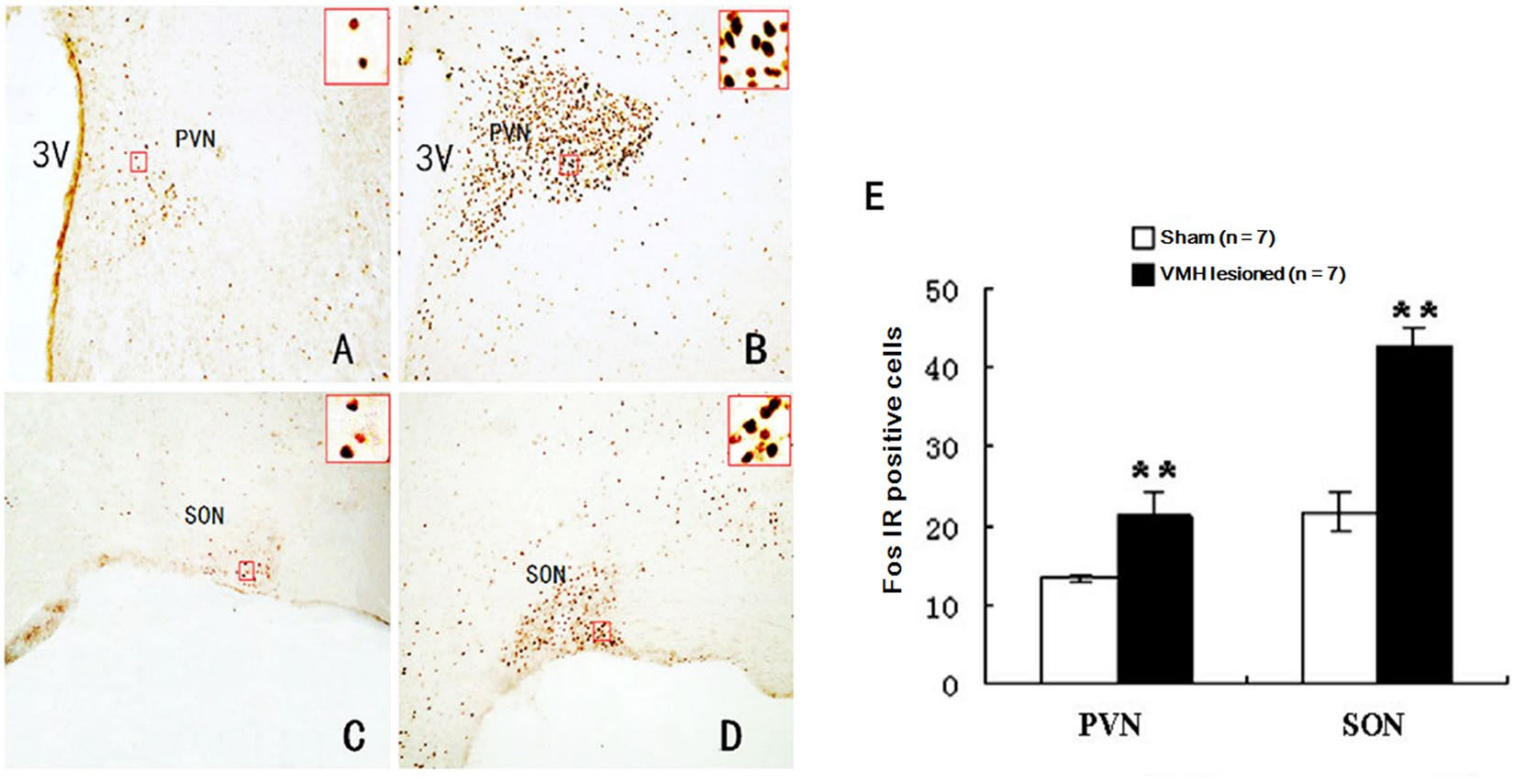
Fos-IR neurons in the PVN and SON of the hypothalamus. (**A**,**C**) Fos-IR neurons in the PVN and SON of the hypothalamus in sham VMH-lesioned RWIS rats. (**B**,**D**) Fos-IR neurons PVN and SON in VMH-lesioned RWIS rats. The insets show higher magnification (×400) of cells in the small boxes. Scale bars = 200 µm. (**E**) Cell counts with immunohistochemical staining of Fos-IR neurons in the PVN and SON of sham VMH-lesioned RWIS rats and VMH-lesioned RWIS rats. Data are presented as the means ± SEM (n = 7). **P < 0.01 compared with the sham group.

**Figure 5:**
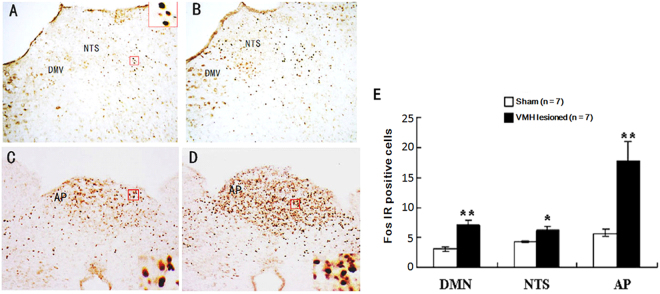
Fos-IR neurons in the DMV, NTS and AP. (**A**,**C**) Fos-IR neurons in the DMV, NTS and AP of the hypothalamus in sham VMH-lesioned RWIS rats. (**B**,**D**) Fos-IR neurons DMV, NTS and AP in VMH-lesioned RWIS rats. The insets show higher magnification (×400) of cells in the small boxes. Scale bars = 200 µm. (**E**) Cell counts with immunohistochemical staining of Fos-IR neurons in the DMV, NTS and AP of sham VMH-lesioned RWIS rats and VMH-lesioned RWIS rats. Data are presented as the means ± SEM (n = 7). **P < 0.01,*P < 0.05 compared with the sham group.

Our previous results showed that RWIS induced obvious Fos expression in the PVN and SON of the hypothalamus and in the DMV, AP, and NTS of the medulla oblongata. In addition, there is a neuroanatomical relationship between the VMH and the PVN, SON, DMV, and NTS. We investigated the impact of VMH lesions on RWIS-induced neuronal activity in the PVN, SON, DMV, NTS and AP. RWIS- induced Fos-positive cells were found in all regions of the VMH-lesioned rats and the sham rats. In the hypothalamus, VMH lesions remarkably increased Fos-positive cells induced by RWIS to 21.30 ± 3.03 in the PVN and 42.65 ± 2.65 in the SON compared with 13.34 ± 0.50 in the PVN and 21.95 ± 2.43 in the SON (Fig. 4A–E). Similarly, in the medulla oblongata, Fos-positive cells within the DMV, NTS and AP were 7.14 ± 0.8, 6.0 ± 0.91, and 7.81 ± 3.20, respectively, in VMH-lesioned rats and showed an obvious increase compared to the sham rats (3.10 ± 0.32 in DMV, 4.38 ± 0.12 in NTS, 5.76 ± 0.58 in AP) (Fig. 5A–E).

## Discussion

Our previous studies have demonstrated that the excitability of VMH neurons may be involved in the stress-induced gastric mucosal injury. The main observations of the present study showed that 1) VMH lesions obviously increased stress-induced gastric mucosal injury, 2) gastric acid and gastric motility in VMH-lesioned rats was significantly enhanced compared to the sham rats, which may have resulted in an increase in gastric erosion induced by RWIS, and 3) VMH lesions remarkably increased Fos-positive cells in the PVN, SON, DMV, NTS and AP induced by RWIS.

RWIS causes gastric mucosal injury due to increased gastric acid secretion and gastric contractility as well as decreased protective factors for gastric mucosa production^19^. The results of our previous studies demonstrated that the NTS, DMV, AP, PVN and SON may be involved in the gastric mucosal injury induced by RWIS^17^. Though the central modulation of gastrointestinal function has been widely studied^20,21^, no study evidence has been reported on the higher central mechanisms of gastric mucosal injury induced by RWIS. Studies from several groups, including our own, have shown that hyperactivity of the vagal parasympathetic nervous system is involved in the gastric mucosal injury induced by RWIS but not the hypothalamo-pituitary-adrenal axis^22^. This finding suggests that neurons in the DMV, NTS and AP were activated following RWIS and cause gastrointestinal dysfunction.

Damage to the ventromedial hypothalamus causes an increase in gastric acid secretion^23,24^. However, the effects of RWIS on gastric acid secretion in VMH-lesioned rats remain unclear. Acid secretion stimulated by 2-DG or insulin is not significantly different between VMH-lesioned rats and control rats^25^. Recent studies demonstrated that gastric acid secretion was nearly three times higher at baseline in VMH-lesioned rats compared to sham VMH-lesioned rats after pentagastrin stimulation^24^. To study the role of the VMH in the genesis of stress-induced gastric mucosal injury, we detected the effect of electrolytic lesions of the VMH on gastric acid secretion and gastric mucosal damage index in rats during water-immersion restraint stress. We found for the first time that water immersion plus restraint stress leads to a remarkable enhancement of gastric acid secretary activity and SGMI in VMH-lesioned rats. During water-immersion restraint stress, a corresponding increase in SGMI in VMH-lesioned rats was associated with an enhancement of acid secretion, indicating a close relationship between acid output and SGMI in VMH-lesioned rats. These results are consistent with the previous findings that the increased acid secretion, the most important aggressive factor, can aggravate stress-induced gastric mucosal injury.

Previous studies have demonstrated that gastric motility was dose-dependently increased following indomethacin administration, and a significant correlation between the lesion index and the changes in gastric motility was found^26^. Our previous studies showed that water-immersion restraint stress resulted in severe gastric mucosal lesions and an increase in gastric motility, and gastric ulceration and motility responses were inhibited by atropine, suggesting that gastric hypermotility may play an important role in the RWIS-induced gastric mucosal injury in rats. The hypermotility and lesion formation in the small intestine induced by indomethacin were obviously inhibited by atropine, implying that hypermotility has an effect on the pathogenesis of small intestinal ulcers induced by non-steroidal anti-inflammatory drugs^27,28^. High-amplitude contractions induced by cold-restraint stress lead to a decrease in mucosal blood flow and attenuate the resistance capacity of gastric mucosa to acute injury. The indomethacin-induced gastric mucosal injury occurred at the top or the bottom of mucosal folding, where the contraction of the stomach resulted in increased gastric mucosal compression and decreased mucosal blood flow^29^. In the present study, both the amplitude and frequency of contractions of the stomach in VMH-lesioned rats significantly increased, and it is most likely that the gastric hypermotility plays a crucial role in aggravating stress-induced gastric injury in VMH-lesioned rats.

Convergent evidence has shown that VMH lesions caused increased mitotic index, thickness of the gastric mucosal cell layer and hypersecretion of gastric acid^24^. Gastric vagotomy restored these parameters to normal. Gastric acid secretion was nearly three times higher at baseline in VMH-lesioned rats compared to sham VMH-lesioned rats after pentagastrin stimulation^23,24^. In addition, recent studies showed that cell proliferation occurred in the visceral organs and could be reversed by subdiaphragmatic vagotomy in the VMH-lesioned rats^30^. A study showed that microinjection of insulin into the VMH altered GI functions by activating the efferent vagus^31^. These results suggest that VMH lesions increase the activities of the vagus nerve, which leads to increased gastric acid secretion and gastric motility.

The SON and PVN locate in the anterior part of the hypothalamus. Recent evidence from our own lab showed that the neurons in SON and PVN were activated and participated in RWIS. A study using an HRP retrograde transport technique found that the VMH receives projection fibers from the SON and PVN. Meanwhile, the PVN receives glutamatergic and GABAergic innervation from the VMH^32,33^. Oxytocin is also released from both parvocellular and magnocellular neurons in the PVN and SON to inhibit food intake by acting on VMH, which contains an abundance of oxytocin receptors^34^. The VMH has an accelerative action in stress-induced hypocalcemia and depressive responses, while the PVN has the opposite effect^15^. These results demonstrated that there exist structural and functional connections between the SON or PVN and the VMH. In the present study, RWIS-induced Fos expression in the PVN and SON in VMH-lesioned rats was obviously higher than that in control rats, suggesting that VMH lesions enhanced neuronal activity in the SON and PVN. The SON and PVN are responsible for the secretion of vasopressin into the general circulation^35^. Vasopressin produces vasoconstriction and significant ischaemia, which may contribute to the gastric mucosal injury in the RWIS rats.

It is possible that there is a different influence on stress-induced gastric ulcers between PVN and VMH. The VMH may have a protective effect against stress-induced gastric ulcers. On the other hand, it is well established that the PVN receives ascending signals from the NTS, and at the same time, it provides direct control over neuronal activities in the DMN and the NTS. Hence, we propose that VMH lesions activated PVN and SON neurons projecting to the DVC in RWIS, which caused vagal-dependent enhancement of acid secretion, motility, and ulcer formation. The DVC is composed of the NTS and the DMN and contains neurons receiving vagal afferent input from the viscera and cell bodies of parasympathetic preganglionic neurons that project to the GI tract. The AP is one of the circumventricular organs of the brain and lacks the normal blood-brain barrier, where circulating hormones enter the brain^36^. Our previous studies demonstrated that RWIS potentiates Fos expression in the DVC, indicating that the DVC plays a key role in stress-induced gastric lesions in rats. Here, we found that RWIS-induced Fos expression in the DMV, NTS and AP in VMH-lesioned rats was clearly higher than that in sham VMH-lesioned rats. Therefore, VMH lesions may enhance DVC neuronal activity, which directly induced the hyperactivity of the parasympathetic nervous system and led to gastric hypercontractility, gastric acid hypersecretion and gastric ulcer formation induced by the stress.

In summary, the present study demonstrated that VMH lesions contributed to increased gastric acid secretion and gastric motility and produced more severe gastric mucosal injury in rats treated with RWIS. The activation of many neurons in the PVN, SON and DVC in VMH-lesioned rats provided neuroanatomical evidence of vagally mediated gastric mucosal injury induced by RWIS. All the results suggest that VMH lesions induced the hyperactivity of the parasympathetic nervous system via an indirect VMH-PVN-DVC-vagal pathway or direct VMH-DVC-vagal pathway and produced gastric dysfunction during RWIS.
